# Metabolomics Analysis for Defining Serum Biochemical Markers in Colorectal Cancer Patients with Qi Deficiency Syndrome or Yin Deficiency Syndrome

**DOI:** 10.1155/2017/7382752

**Published:** 2017-07-24

**Authors:** Fangfang Tao, Ping Lü, Chunbo Xu, Mengmeng Zheng, Wenhong Liu, Minhe Shen, Shanming Ruan

**Affiliations:** ^1^Department of Immunology and Microbiology, Basic Medical College, Zhejiang Chinese Medical University, Hangzhou, Zhejiang, China; ^2^Department of Traditional Chinese Medicine, Taizhou First People's Hospital, Taizhou, Zhejiang, China; ^3^Department of Internal Medicine, Yuyao Hospital of Traditional Chinese Medicine, Yuyao, Zhejiang, China; ^4^Department of Medical Oncology, Wenzhou Hospital of Integrated Traditional Chinese and Western Medicine, Wenzhou, Zhejiang, China; ^5^Department of Medical Oncology, The First Affiliated Hospital, Zhejiang Chinese Medical University, Hangzhou, Zhejiang, China

## Abstract

Colorectal cancer is one of the leading causes of tumor-associated death, and traditional Chinese medicine (TCM) classifies colorectal cancer into various subtypes mainly according to the symptomatic pattern identification* (ZHENG)*. Here, we investigated the difference in metabolic profiles of serum by comparing colorectal cancer subjects with Nondeficiency (ND), Qi deficiency (QD), and Yin deficiency (YD). The ratio of subjects with carcinoembryonic antigen (CEA) was higher in YD pattern, and the ratio of subjects with carbohydrate antigen 19-9 (CA19-9) was higher both in YD and in QD, compared with ND. As a result of metabolomics analysis, twenty-five metabolites displayed differences between QD and ND, while twenty-eight metabolites displayed differences between YD and ND. The downregulated metabolites in QD/ND and YD/ND mainly include carbohydrates and the upregulated metabolites mainly include amino acids and fatty acids, suggesting conversion obstruction of carbohydrates, fatty acids, and amino acids occurs in patients with QD and YD compared with ND. Our results demonstrate that colorectal cancer patients with QD or YD were associated with metabolic disorders and the variations of serum metabolic profiles may serve as potential biochemical markers for diagnosis and prognosis of colorectal cancer patients displayed QD or YD patterns.

## 1. Introduction

Colorectal cancer (CRC) is globally one of the most commonly diagnosed cancers, which is the fourth leading cause of death in cancer patients [1]. At present, a combination of radiotherapy and chemotherapy is the top choice for CRC treatment [2]. However, the prognosis and survival rate of patients with advanced CRC are poor due to high postsurgical tumor metastasis. The common chemotherapeutic drugs used to treat CRC include 5-Fluorouracil/Leucovorin (5FU/LV), capecitabine, oxaliplatin, irinotecan [2]. Serious side effects caused by these therapies promote us to find alternative therapies with high efficacy but few side effects.

TCM, which emphasizes bringing the patient's body, mind, and spirit into harmony, is coming to a promising and alternative approach for the prevention and treatment of tumor patients including CRC. TCM rests squarely on* ZHENG* (syndrome) differentiation, a process of analyzing data collected through four combined diagnostic methods:* WANG* (inspection),* WEN* (falling-rising tone, auscultation, and olfaction),* WEN* (falling tone, inquiry), and* QIE* (palpation). All diagnostic and therapeutic methods in TCM are based on the differentiation of* ZHENG*. According to the theory of TCM, patients with a specific disease, including cancer, also exhibit various types of syndrome* (ZHENG)* and categorization of different types of syndrome is a critical concept to recognize the nature of cancer patients. Treatment of cancer based on* ZHENG* differentiation, also known as* “Bian Zheng Lun Zhi,”* can be used to guide the choice of treatment with TCM herbal formulae. In recent years, there was a dramatic increase in the total number of publications reporting the concept of TCM* ZHENG* in the cancer therapy.

Qi deficiency (QD) and Yin deficiency (YD) are two common syndromes in CRC patients. Qi refers to the vital energy of the body in TCM. It maintains blood circulation, warms the body, and fights diseases. Qi deficiency is the most common symptom in cancer patients according to the concept of TCM. Many previous reports showed that Qi supplementation can help enhance the effects of cancer therapy and the main role of TCM in cancer therapy is to balance the Qi flow in cancer patients. Yin deficiency usually represents a status of the human body under lack of nutrition and fluid and usually manifests as emaciation, dizziness, vertigo, tinnitus, dryness of the mouth, fever, and night sweats [3–5]. It should be of great importance to examine how Qi deficiency and Yin deficiency affect CRC.

As an important component of systems biology, metabolomics is the study of small biological molecules found within cells, tissues, and body fluids in response to environmental, pathogenic, and dietary changes or a genetic alteration and aims to characterize and quantify all the small compounds in complex biological samples. Metabolomics, as well as genomics and proteomics, has been used to identify candidate biomarkers closely related to pathological processes of diseases [6–9]. It can help us to discover the mechanism of disease formation and progression. Some metabolomics studies have been applied to cancer patients. For instance, Ma et al. reported 10 potential oncofetal biomarkers and validated their potential for CRC diagnosis. Chen et al. showed that metabolomic profiling approach is a promising screening tool for the diagnosis and stratification of human hepatocellular carcinoma.

In this study, metabolomics profiling was performed by using GC-MS to compare the difference of serum metabolic profiles in colorectal cancer subjects with ND, QD, and YD and our results demonstrate that colorectal cancer patients with QD or YD were associated with metabolic disorders and the variations of serum metabolic profiles may serve as potential biochemical markers for diagnosis and prognosis of colorectal cancer patients displayed QD or YD patterns.

## 2. Materials and Methods

### 2.1. Study Subjects

This research protocol was approved by the local medical ethics committee of Zhejiang Chinese Medical University and registered in Chinese Clinical Trial Registry (registration number: ChiCTR-OCH-13003261). A total of 90 CRC patients were consecutively recruited from July 2013 to July 2014 in Hangzhou, Zhejiang, China. All subjects were genetically unrelated ethnic Han Chinese.

### 2.2. Diagnostic Criteria

Diagnoses of all of the patients were confirmed by pathology. Trained interviewers used a uniform questionnaire to collect the TCM diagnostic information from the participants, namely, demographic factors such as age and gender, and known risk factors for CRC (including drinking, diet habit, individual disease history, marriage, and birth history). The standard criteria used for classification of CRC ZHENG were as described previously [10]. Three types of CRC ZHENG were used: Qi deficiency syndrome, Yin deficiency syndrome, and no deficiency syndrome. Since many factors may affect the formation of TCM syndromes, more than one TCM syndrome was observed in the majority of patients. To ensure a uniform and standard CRC ZHENG, the most significant TCM syndromes functioned as units, which were worked out concurrently by two TCM clinical experts.

### 2.3. Inclusion Criteria

Advanced colorectal cancer patients meet criterions of western medicine and TCM and the following characteristics were included in the study: (a) aged between 18 and 75 years, (b) Han Chinese ethnicity, (c) newly histopathologically diagnosed with primary CRC, (d) lack of previous malignant tumors in other organs, (e) had not had antitumor therapy before recruitment, including chemotherapy and radiotherapy, and (f) did not have severe heart failure, pulmonary insufficiency, or kidney disease.

### 2.4. Exclusion Criteria

Patients with jejunum tumor, appendix tumor, colorectal adenoma, E. stromal tumor, large intestine malignant melanoma, and large intestine leiomyosarcoma and cases without pathological diagnosis and completed data were excluded.

### 2.5. CRC Sample Preparation

CRC serum samples were purified through centrifugation of blood (3000 rpm, 10 min, and 25°C). Supernatant was collected and stored at −20°C until further analysis. Prior to GC-MS analysis, 1 mL of cold methanol was added to 100 *μ*L of serum and then vortex mixed for 1 min. 10 *μ*L of L-phenylalanine was added as internal standard. The sample mixture was then centrifuged at 3000 rpm for 15 min at 4°C. 200 *μ*L of supernatant was blown to dryness under a gentle nitrogen flow. Then, samples were derivatized by 30 *μ*L methoxyamine hydrochloride (20 mg/mL in pyridine, 2 h, 37°C) and 30 *μ*L N,O-bis(trimethylsilyl)-trifluoroacetamide (MSTFA) (1% N-Trimethysilylimidazole included, 1 h, 70°C), for GC-MS analysis.

### 2.6. GC-MS Analysis of CRC Serum Samples

One microliter of each sample was injected into the GC (Agilent 7890A/5975C) system in the splitless mode. GC separation was conducted on a capillary column HP-5MS (30 m × 0.25 mm × 0.25 *μ*m, Agilent J&W Scientific, USA). The injector temperature was controlled at 280°C and the split rate of the injector was 1 : 50. Helium was used as a carrier gas at a constant flow rate of 1.0 mL/min. The initial column temperature was kept at 80°C for 2 min, and then the temperature was increased to 320°C at a rate of 10°C/min and held there for 6 min. The ion-source temperature was controlled at 230°C. Mass spectra were recorded from* m*/*z* 50 to 550 at a rate of 2 s in full-scan mode, and the solvent delay time was 3 min.

### 2.7. Data Processing and Multivariate Data Analysis

The GC-MS data was processed using the automatic mass spectral deconvolution and identification system (AMDIS, version 2.71) and the metabolomics ion-based data extraction algorithm (MET-IDEA, version 2.08). Multivariate data analysis was achieved on the normalized GC-MS datasets with software package SIMCA-P (version 13.0, Umetrics, Sweden). Principal component analysis (PCA) was carried out on the dataset to generate an overview of the sample distribution and observe possible outliers. The partial least-squares discrimination analysis (PLS-DA) was further performed with the unit-variance scaled GC-MS data as *X* matrix and class information as *Y* matrix to identify the metabolites that significantly contribute to intergroup differentiation. The PLS-DA models were validated using a sevenfold cross validation method and the quality of the model was described by the parameters of* R*^2^*X* and* Q*^2^ values. The Variable Importance in the Projection (VIP) value (VIP > 1) was used to evaluate the variable contribution and identify the potential biomarkers. Metabolite set enrichment analysis was performed by using online software MetaboAnalyst (http://www.metaboanalyst.ca/).

### 2.8. Statistical Analysis

The univariate statistical analysis was performed by SPSS 19.0 for further identification of potential biomarkers, including box figure analysis and analysis of variance (ANOVA), and *P* value was set as 0.05 for statistical significance.

## 3. Results

### 3.1. Association of the QD and YD Subtypes of CRC Samples with Higher Levels of CEA and CA199

A total of 90 patients with stage III-IV CRC were subjected to perform GC-MS, 30 samples for each group. Before GC-MS analysis, the association of QD and YD subtypes with patient clinicopathological characteristics was calculated. The general clinicopathological characteristics are shown in Table 1, including gender, primary occurrence site, tumor stage, alanine aminotransferase (ALT), aspartate transaminase (AST), total bilirubin (TBIL), direct bilirubin (DBIL), serum creatinine (Scr), blood urea nitrogen (BUN), carcinoembryonic antigen (CEA) and carbohydrate antigen 19-9 (CA199). The YD subtype in CRC had a significant association with higher CEA and CA199 expression compared with the ND and QD group (Table 1).

### 3.2. PCA and PLS-DA Analysis of Metabolomics Profiles in the Three Groups (ND; QD; YD) of CRC Patients

**Figure 1:**
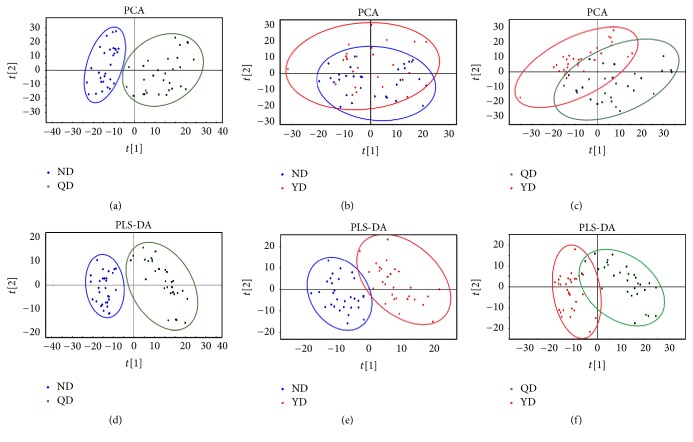
PCA and PLS-DA scores plots discriminating CRC serum samples with QD or YD from those with ND. (a) PCA: QD versus ND; (b) PCA: YD versus ND; (c) PCA: QD versus YD. (d) PLS-DA: QD versus ND; (e) PLS-DA: YD versus ND; (f) PLS-DA: QD versus YD.

Principal component analysis (PCA) was used to determine the presence of inherent similarities in spectral profiles and the corresponding PLS-DA analysis was used to identify discriminating metabolites and differentiate the two groups. PCA and PLS-DA applied to the differentially expressed metabolites (*P* < 0.05) revealed a clear separation of the QD and ND samples (Figures 1(a) and 1(d)), which could be attributed to differential metabolites. There was no statistically significant difference in expression values between QD and YD samples, while either PCA or PLS-DA analyses were applied (Figures 1(c) and 1(f)). For groups of YD and ND, although PCA results partially overlapped, the PLS-DA loading plot showed that the distribution of the two groups differed (Figures 1(b) and 1(e)). These results demonstrated that the difference of plasma biological signatures between QD and ND was more significant than those between YD and ND, which were in accordance with the concept that YD are attributed to metabolic disorders.

### 3.3. Differentially Expressed Metabolite Identification and the Potentially Related Pathway among the QD, YD, and ND Samples

**Figure 2:**
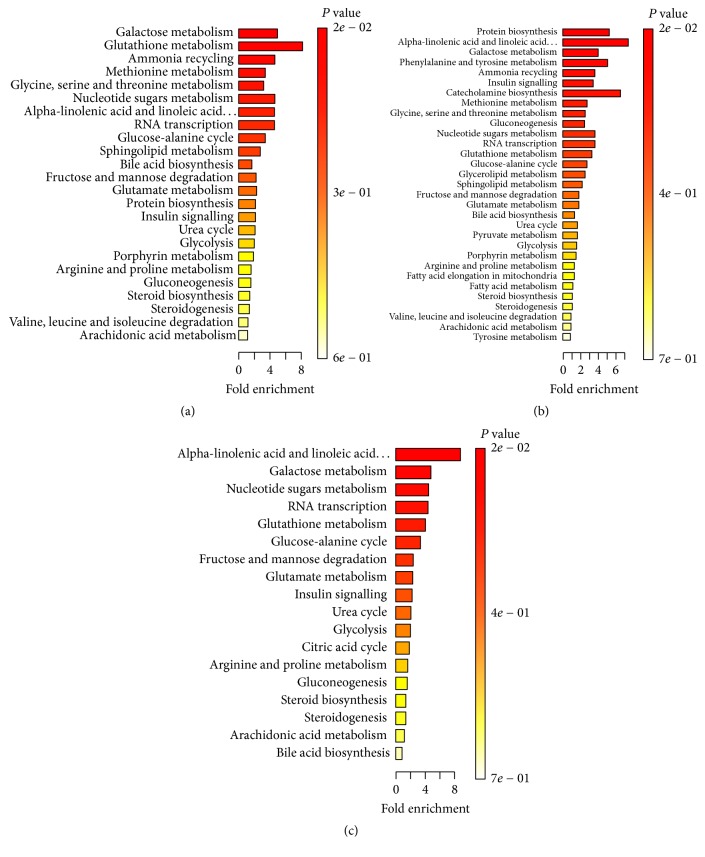
Metabolite set enrichment analysis (MSEA) to capture the metabolomic diversity of serum samples of CRC patients with QD or YD compared with those with ND. (a) QD versus ND; (b) YD versus ND; (c) QD versus YD.

**Figure 3:**
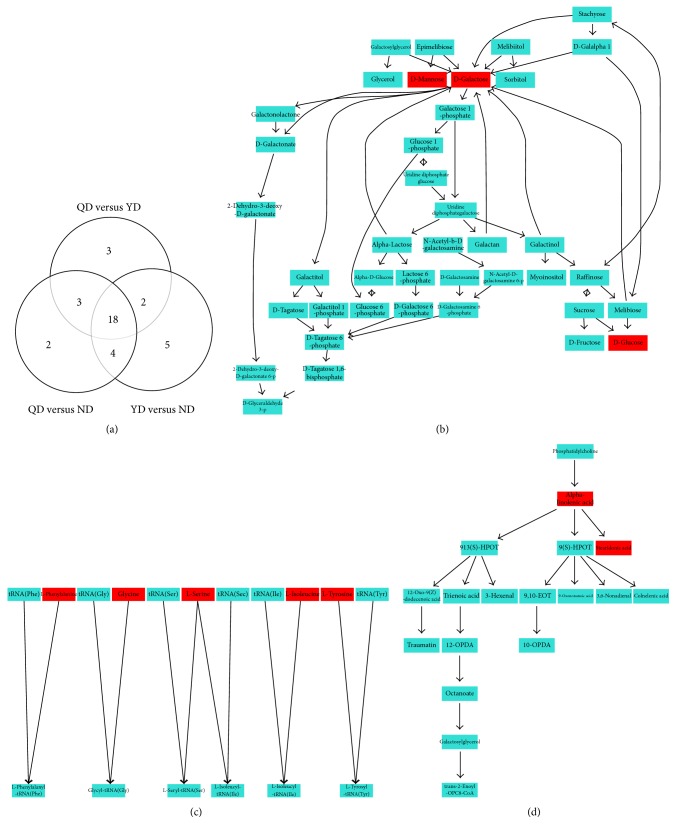
Different metabolic pathways were strongest affected by QD or YD in CRC serum samples. (a) Venn diagram showing overlap among metabolites differentially expressed in serum samples of CRC patients with QD, YD, or ND. (b, c, and d) Schematic representation of galactose metabolism (b), tRNA synthesis (c), and alpha-linolenic acid metabolism (d) pathway. In red, the metabolites that differentially expressed in serum samples of CRC patients with QD, YD, or ND.

**Figure 4:**
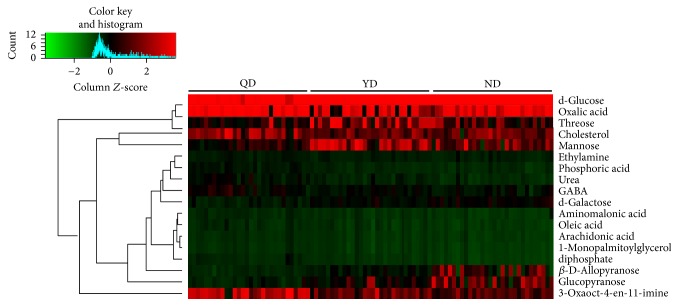
Heatmap is shown from hierarchical clustering analyses of metabolomics changes in serum samples of CRC patients with QD, YD, or ND.

For QD versus ND, a total of 27 discriminating metabolites (VIP > 1.0, *P* < 0.05), including 21 in positive mode and 6 in negative mode (Table 2), were identified in plasma. These results showed that most metabolites increased in QD samples, suggesting accelerated metabolism processes in QD patients. For YD versus ND, we also identified 29 discriminating metabolites, including 23 in positive mode and 6 in negative mode (Table 3). For QD versus YD, 26 discriminating metabolites were identified, including 19 in positive mode and 7 in negative mode (Table 4). Most metabolites increased in QD or YD patients with CRC. The possible pathways related to the conditions under study were identified with MetaboAnalyst 3.0, a free online tool based on the high-quality KEGG metabolic pathways database. The pathway impact value was calculated from pathway topology analysis. For QD versus ND, the top potential pathways were galactose metabolism, (Figures 2(a) and 3(b)). For YD versus ND, the top three potential pathways were protein biosynthesis (Figures 2(b) and 3(c)). For QD versus YD, the top three potential pathways were linolenic acid metabolism (Figures 2(c) and 3(d)). Among the differential metabolites among QD, YD, and ND samples, we found that 18 metabolites appeared in Tables 2 and 3 (Figure 3(a)) at the same time. Hierarchical clustering is commonly used for unsupervised clustering. The results showed that CRC patients with QD, YD, or ND syndrome could be distinguished well (Figure 4).

## 4. Discussion

Traditional Chinese medicine (TCM) has been widely used to relieve the symptom of colorectal cancer. Chinese medicine syndrome (CMS) is an understanding of the regularity of disease occurrence and development and correct classification of CMS groups is very important as all diagnostic and therapeutic methods in TCM are based on TCM syndrome groups. However, it is difficult to decipher the scientific basis and systematic features of CMS as of the complexity of CMS and the limitation of the present investigation method. Metabolomics enables mapping of early biochemical changes in disease and hence provides a useful tool to develop predictive biomarkers. Moreover, its method itself resembles traditional Chinese medicine (TCM) that focuses on human disease via the integrity of close relationship between the human body, fluids, and syndromes. Systemically, metabolomics has a convergence with TCM syndrome and therefore provides useful methods for exploring the essence of CMS, facilitating personalized treatment with TCM. Importantly, the integration of metabolomics and CMS will bridge the gap between Chinese and Western medicine. In the present study, we employed GC-MS to compare metabolomic profiles in serum samples of CRC patients with QD, YD, and ND. Distinctly different metabolic patterns were observed among the 3 groups. Our results suggest that a panel of unique serum metabolites is clinical potential biomarker set for the disease diagnosis and CMS classification for CRC patients. These metabolite markers would give a promise to reflect the essence of the patients with QD or YD. Moreover, the energy metabolism disorder is specially prominent in CRC patients with QD, while the process of protein synthesis is more seriously disordered in those with YD, which is in accordance with the traditional theory of TCM for Qi and Yin deficiency [11, 12].

To investigate colorectal cancer metabolism, Zhang et al. performed an electronic literature search, from 1998 to January 2016 to evaluate the metabolomic profile of patients with CRC regarding the diagnosis, recurrence, and prognosis/survival and systematically review the twenty-three literatures included [13]. They identified the most important biomarkers in CRC related to carbohydrate, lipid, amino acid, nucleotide, and other significant metabolites. Among them, some metabolites were also identified to be deregulated in our studies. For instance, we found that d-galactose was downregulated in YD and QD patients compared with ND, especially in QD. This metabolite was also shown to be downregulated in CRC patients in Zhang et al.'s review. These results demonstrate that the level of d-galactose in serum may be a specific biomarker to classify CRC with different deficiency syndrome.

Metabolomic data typically contains lots of variables, which are interrelated. Multivariate statistical methods such as PCA and OPLS-DA coupled with univariate statistical methods such as Student's *t*-test were used in this study. Our study revealed different metabolic pathways associated with QD and YD in CRC patients via GC-MS. PCA and PLS-DA plots differed among plasma of CRC patients with QD, those with YD, and those with ND, which indicates the presence of different metabolites. For example, our data demonstrate the metabolite urea was upregulated in CRC patients with YD and QD, especially in samples with QD. This result suggests that amino acid metabolism may play a vital role in classification of these samples. However, this metabolite was reported to be decreased in CRC cases in all studies [14, 15]. In our future study, we aim to study the precise role of urea in CRC patients with YD and QD. Meanwhile, our results demonstrate that the YD group is more strongly overlapping with the ND group compared with the QD group, suggesting that CRC patients with QD may display more severely metabolic disorder during cancer occurrence and progression.

On the other hand, in this study, 24 metabolic pathways related to 27 discriminating metabolites were found in the QD group compared with the ND group, while 31 metabolic pathways related to 27 discriminating metabolites were found in the YD group compared with the ND group. These results indicate that although more severely metabolic disorder occurs during cancer occurrence and progression in CRC patients with QD, YD influences more metabolic pathways in a weaker level. This phenomenon could offer a possible explanation for the reason why CRC patients with YD were more difficult to treat in some extent [3, 4].

One of the limitations of our study was insufficient samples. Only 30 samples were included for each group, which is a small number for such a complicated disease and syndrome. A study on a larger scale should be conducted to establish a precise metabolomics diagnostic model.

Ma et al. developed an integrated proteomics and metabolomics approach for defining oncofetal biomarkers in the colorectal cancer and 5 individual metabolites and the 5 individual proteins were characterized and their potential for CRC diagnosis was validated [16]. Another limitation of this study was no explanation was offered to demonstrate the reason why these metabolites are changed in CRC patients with QD or YD. To verify the upstream changes in metabolites, further studies must be conducted using proteomics and transcriptomics.

## Figures and Tables

**Table 1:** Clinicopathological characteristics of studied patients.

Characteristics	Subtypes of colorectal cancer			*P* value
	ND (*n* = 30)	QD (*n* = 30)	YD (*n* = 30)	
Sex				
Male	18	18	20	>0.05
Female	12	12	10	
Primary site				
Colon	18	22	18	>0.05
Rectum	12	8	12	
Tumor stage (pTNM)				
III	14	16	13	>0.05
IV	16	14	17	
ALT				
Normal	28	25	28	>0.05
High	2	5	2	
AST				
Normal	29	24	26	>0.05
High	1	6	4	
TBIL				
Normal	26	29	26	>0.05
High	4	1	4	
DBIL				
Normal	26	29	26	>0.05
High	4	1	4	
Scr				
Low	3	6	8	>0.05
Normal	27	24	21	
High	0	0	1	
BUN				
Low	2	2	1	>0.05
Normal	27	28	28	
High	1	0	1	
*CEA*				
* Normal*	*21*	*22*	*12*	*<0.05*
* High*	*9*	*8*	*18*	
*CA199*				
* Normal*	*26*	*21*	*11*	*<0.05*
* High*	*4*	*9*	*19*	

All *P* values, except those for Scr and BUN, were calculated by using the Chi-square test. *P *values for Scr and BUN were calculated by using Kruskal-Wallis's exact test.

**Table 2:** Differential metabolites in the plasmas of CRC patients with QD compared with those with ND.

	VIP	*mz*	rt	Name	*t*-test	log_2_(QD/ND)
Up-regulated	1.176	451	14.43	Diphosphate	0.000	4.861
	1.432	248	9.97	Glycine	0.000	2.431
	1.675	80	20.93	Arachidonic acid	0.000	2.236
	1.732	79	9.27	Urea	0.000	2.193
	1.361	157	12.70	Pyroglutamic acid	0.000	2.101
	1.031	137	19.63	Oleic acid	0.000	2.031
	1.37	258	7.43	3-Oxaoct-4-en-11-imine	0.000	2.002
	1.341	248	12.07	Aminomalonic acid	0.000	1.939
	1.099	432	18.81	Inositol	0.000	1.907
	1.18	299	9.77	L-Isoleucine	0.000	1.898
	1.324	188	10.66	Serine	0.000	1.783
	1.557	370	22.69	Monopalmitoylglycerol	0.000	1.711
	1.638	181	9.64	Phosphoric acid	0.000	1.659
	1.102	77	6.22	Carbamate	0.000	1.585
	1.131	170	6.52	Hydroxycyclohexane	0.000	1.559
	1.172	84	16.32	N-*α*-Acetyl-L-Lysine	0.000	1.446
	1.414	133	7.73	Oxalic acid	0.000	1.415
	1.567	155	11.92	GABA	0.000	1.359
	1.526	57	27.67	Cholesterol	0.000	1.154
	1.543	155	11.58	Ethylamine	0.000	0.777
	1.168	217	17.22	Allose	0.000	0.737

Down-regulated	1.265	73	17.06	d-Glucose	0.000	−0.414
	1.621	75	17.06	Threose	0.000	−1.296
	1.38	321	16.98	d-Galactose	0.000	−1.813
	1.652	221	17.04	Mannose	0.000	−1.815
	1.191	205	16.89	Glucopyranose	0.000	−2.039
	1.34	206	17.70	*β*-D-Allopyranose	0.000	−2.316

**Table 3:** Differential metabolites in the plasmas of CRC patients with YD compared with those with ND.

	VIP	*mz*	rt	Name	* t*-test	log_2_(YD/ND)
Upregulated	2.176	451	14.43	Diphosphate	0.000	2.926
	1.7	299	9.77	L-Isoleucine	0.000	1.946
	1.635	248	9.97	Glycine	0.000	1.818
	1.29	75	6.63	Lactic acid	0.007	1.319
	1.619	80	20.93	Arachidonic acid	0.001	1.292
	1.716	188	10.66	Serine	0.000	1.141
	1.612	370	22.69	1-Monopalmitoylglycerol	0.001	1.046
	1.563	60	7.73	Oxalic acid	0.001	1.033
	1.567	248	12.07	Aminomalonic acid	0.001	0.973
	1.295	84	16.32	N-*α*-Acetyl-L-lysine	0.006	0.877
	1.24	137	19.63	Oleic acid	0.009	0.818
	1.527	79	9.27	Urea	0.001	0.799
	1.061	192	13.92	Phenylalanine	0.027	0.679
	1.112	342	19.83	Stearic acid	0.020	0.674
	1.945	258	7.43	3-Oxaoct-4-en-11-imine	0.000	0.568
	1.029	179	16.66	Tyrosine	0.032	0.533
	1.576	155	11.92	GABA	0.001	0.510
	1.119	132	9.22	5-Hydroxycaproic acid	0.020	0.497
	1.018	97	18.08	Palmitic acid	0.034	0.462
	1.261	181	9.64	Phosphoric acid	0.008	0.380
	1.16	79	19.59	Linoleic acid	0.015	0.368
	1.23	57	27.67	Cholesterol	0.010	0.329
	1.164	155	11.58	Ethylamine	0.015	0.317

Downregulated	1.095	73	17.06	d-Glucose	0.022	−0.159
	1.079	75	17.06	Threose	0.025	−0.279
	1.161	321	16.98	d-Galactose	0.015	−0.517
	1.41	221	17.04	Mannose	0.003	−0.548
	1.19	205	16.89	Glucopyranose	0.013	−0.780
	1.754	206	17.70	*β*-D-Allopyranose	0.000	−1.190

**Table 4:** Differential metabolites in the plasmas of CRC patients with QD compared with those with YD.

	VIP	*mz*	rt	Name	*t*-test	log_2_(QD/YD)
Upregulated	1.181	451	14.43	Diphosphate	0.001	1.935
	1.089	299	15.47	Phosphoric acid propyl ester	0.001	1.728
	1.522	258	7.43	3-Oxaoct-4-en-11-imine	0.000	1.434
	1.714	79	9.27	Urea	0.000	1.395
	1.878	181	9.64	Phosphoric acid	0.000	1.279
	1.01	137	19.63	Oleic acid	0.003	1.213
	1.2	170	6.52	Hydroxycyclohexane	0.000	1.113
	1.107	157	12.70	Pyroglutamic acid	0.001	1.045
	1.036	77	6.22	carbamate	0.003	0.999
	1.202	273	16.06	Citric acid	0.000	0.992
	1.17	248	12.07	Aminomalonic acid	0.001	0.966
	1.466	80	20.93	Arachidonic acid	0.000	0.944
	1.028	79	19.59	Linoleic acid	0.003	0.904
	1.098	133	7.73	Oxalic acid	0.001	0.888
	1.598	155	11.92	GABA	0.000	0.849
	1.665	57	27.67	Cholesterol	0.000	0.825
	1.274	370	22.69	1-Monopalmitoylglycerol	0.000	0.665
	1.203	341	19.82	Stearic acid	0.000	0.626
	1.261	155	11.58	Ethylamine	0.000	0.461

Downregulated	1.04	73	17.06	d-Glucose	0.002	−0.255
	1.759	75	17.06	Threose	0.000	−1.017
	1.561	206	17.70	*β*-D-Allopyranose	0.000	−1.126
	1.335	205	16.89	Glucopyranose	0.000	−1.259
	1.622	221	17.04	Mannose	0.000	−1.267
	1.924	321	16.98	d-Galactose	0.000	−1.296
	1.668	132	9.22	5-Hydroxycaproic acid	0.000	−1.482

## Abbreviations

TCM: Traditional Chinese medicine
CMS: Chinese medicine syndrome
CRC: Colorectal cancer
QD: Qi deficiency
YD: Yin deficiency
ND: Nondeficiency
CEA: Carcinoembryonic antigen
CA19-9: Carbohydrate antigen 19-9
ALT: Alanine aminotransferase
AST: Aspartate transaminase
TRIL: Total bilirubin
DBIL: Direct bilirubin
Scr: Serum creatinine
BUN: Blood urea nitrogen
GC-MS: Gas chromatography–mass spectrometry
PCA: Principal component analysis
PLS-DA: Partial least-squares discrimination analysis
VIP: Variable Importance in the Projection.
